# Micronutrient Networks in Nutritional Anemia: A Narrative Review Beyond Pathophysiology, Diagnosis, and Management of Iron Deficiency

**DOI:** 10.7759/cureus.113205

**Published:** 2026-07-23

**Authors:** Shridhar Chougule, Malavika T, Amar Mohite, Vinod Choudhary, Sivanathan Thangvelu, Kailas Datkhile, Jayant Pawar

**Affiliations:** 1 Research, Krishna Vishwa Vidyapeeth (Deemed to be University), Karad, IND; 2 Biotechnology, Krishna Institute of Science and Technology, Krishna Vishwa Vidyapeeth (Deemed to be University), Karad, IND; 3 Pediatrics, Krishna Institute of Medical Sciences, Krishna Vishwa Vidyapeeth (Deemed to be University), Karad, IND; 4 Pediatrics and Child Health, Orange City Institute of Nursing, Warud, IND

**Keywords:** adolescent nutrition, anemia mukt bharat, erythropoiesis, folate, iron deficiency, micronutrient interactions, micronutrients, nutritional anemia, precision nutrition, vitamin b12

## Abstract

Nutritional anemia remains one of the most persistent global health disorders, affecting populations across all age groups, particularly women, children, and individuals from low- and middle-income countries. Although iron deficiency has traditionally been considered the principal cause of nutritional anemia, emerging data indicate that anemia frequently results from intricate interactions among multi-micronutrient deficiencies, including folate, vitamin B12, vitamin A, zinc, copper, selenium, and antioxidant vitamins. Micronutrients regulate erythropoiesis, iron homeostasis, DNA synthesis, oxidative balance, and red blood cell survival.

Recent advances in nutritional and molecular research indicate that micronutrient deficiencies frequently occur in combinations rather than as isolated abnormalities. Instead, synergistic and antagonistic interactions among micronutrients may influence disease severity and treatment response, and diagnostic interpretation. Furthermore, inflammation, gut dysbiosis, infections, dietary inhibitors, and genetic variability further complicate micronutrient metabolism and iron utilization.

This review critically examines the combined role of micronutrients in the pathophysiology of nutritional anemia, with emphasis on erythropoietic regulation, iron metabolism, oxidative stress, and micronutrient cross-talk. The review also discusses current diagnostic challenges, emerging biomarkers, personalized nutrition approaches, and public health strategies aimed at improving anemia management.

All the evidence converges toward a shift from an iron-centric approach to a broader multi-micronutrient framework for the management of nutritional anemia.

## Introduction and background

Nutritional anemia is a multifactorial hematological disorder characterized by reduced hemoglobin concentration or impaired oxygen-carrying capacity resulting from deficiencies of essential nutrients required for erythropoiesis [1,2]. It continues to represent a major global public health challenge, affecting almost one-fourth of the world's population, with the highest burden observed among pregnant women, children, adolescents, and socioeconomically vulnerable populations. The prevalence remains disproportionately high in low- and middle-income countries, where inadequate dietary intake, recurrent infections, chronic inflammation, and poor healthcare accessibility coexist.

Historically, nutritional anemia has been predominantly associated with iron deficiency [2,3]. Consequently, most clinical and public health interventions have focused primarily on iron supplementation and food fortification. However, increasing evidence indicates that anemia frequently develops due to concurrent deficiencies of multiple micronutrients, including folate, zinc, copper, selenium, vitamin B12, A, and C [4-7]. These micronutrients are involved in diverse yet interconnected physiological pathways regulating erythropoiesis, DNA synthesis, heme production, iron transport, mitochondrial metabolism, and oxidative stress regulation.

Recent studies further suggest that micronutrient deficiencies rarely occur independently [8,9]. Instead, overlapping interactions among nutrients significantly influence their absorption, transport, and biological utilization. For example, excessive zinc intake may impair copper absorption, while vitamin C enhances non-heme iron bioavailability. Similarly, chronic inflammation and infection alter iron homeostasis through hepcidin-mediated sequestration, thereby contributing to functional iron deficiency even though adequate iron reserves are present.

Although iron deficiency anemia (IDA) has been widely studied, the combined contribution of other micronutrients to anemia remains less clearly addressed [10,11]. Most conventional diagnostic and therapeutic approaches continue to rely on isolated nutrient assessment, often overlooking mixed deficiency states and subclinical micronutrient imbalances. This limitation contributes to underdiagnosis, poor treatment response, and persistent disease burden.

Therefore, this review aims to provide an exhaustive and mechanistic outline of micronutrient-associated nutritional anemia by integrating current knowledge on erythropoiesis, iron metabolism, oxidative stress, nutrient interactions, diagnostic challenges, therapeutic strategies, and precision nutrition. Unlike conventional discussions that focus mainly on iron deficiency, this review highlights the broader network of micronutrients involved in anemia, including their interactions with inflammation, gut absorption, oxidative stress, and mixed-deficiency states. Such an approach may help clinicians recognize mixed deficiencies more effectively and avoid relying solely on iron replacement.

Methodology/literature search strategy

This narrative review was prepared by examining published literature related to nutritional anemia, micronutrient deficiencies, erythropoiesis, iron metabolism, diagnostic biomarkers, and anemia management. Appropriate articles were explored through PubMed, Scopus, Web of Science, Google Scholar, and official public health sources, such as the World Health Organization and national health program documents.

The search terms included “nutritional anemia”, “iron deficiency anemia”, “micronutrient deficiency”, “vitamin B12 deficiency”, “folate deficiency”, “vitamin A and anemia”, “zinc and anemia”, “copper deficiency anemia”, “selenium and erythropoiesis”, “vitamin C and iron absorption”, “hepcidin”, “functional iron deficiency”, “micronutrient interactions”, and “multi-micronutrient supplementation”. Original research articles, review articles, clinical studies, meta-analyses, and public health reports were considered. Greater emphasis was given to studies discussing the biological role of micronutrients in red blood cell formation, iron transport, oxidative stress, inflammation, diagnosis, and therapeutic strategies.

Articles not directly related to nutritional anemia, micronutrient-associated hematological changes, or anemia-related public health interventions were excluded. Since this is a narrative review, formal meta-analysis or quantitative evidence grading was not performed. Instead, the available literature was synthesized to provide a broad mechanistic and clinical understanding of nutritional anemia beyond iron deficiency.

## Review

Role of micronutrients in hematopoiesis

**Figure 1:**
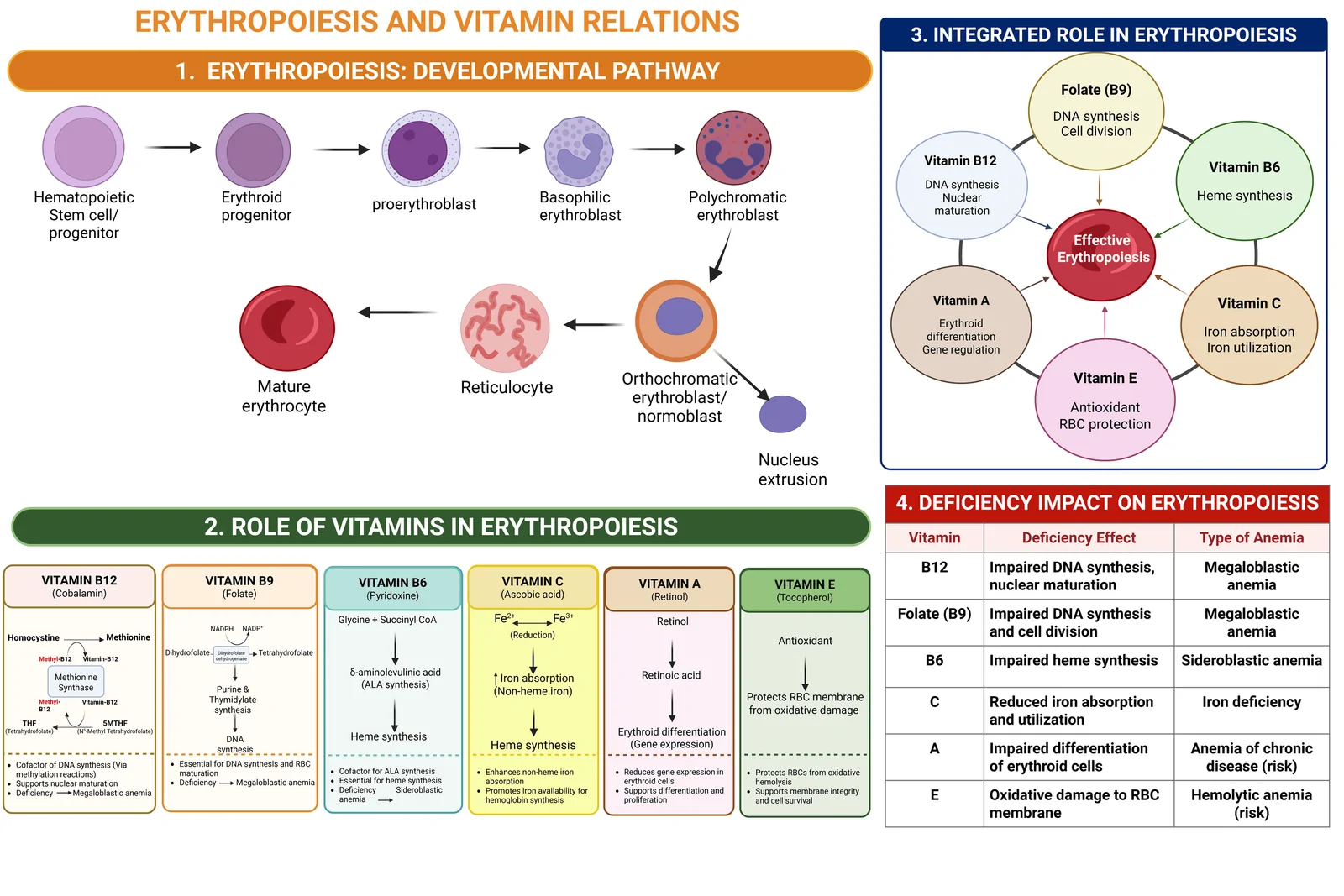
Role of vitamins in erythropoiesis and red blood cell maturation. This image illustrates the developmental pathway of erythropoiesis and highlights the contribution of key vitamins, including vitamin B12, B9, B6, C, A, and E, in DNA synthesis, heme formation, iron absorption, erythroid differentiation, and red blood cell protection. Image Credit: This figure was created using BioRender (BioRender.com, Toronto, ON, Canada).

Hematopoiesis is a strictly coordinated physiological process that involves the proliferation, differentiation, and maturation of hematopoietic stem cells within the bone marrow. Erythropoiesis, the process of red blood cell formation, is particularly dependent on adequate availability of micronutrients that regulate DNA synthesis, mitochondrial metabolism, heme biosynthesis, enzymatic activity, and oxidative balance (Figure 1).

Iron plays a key role in erythropoiesis as a crucial component of heme, the oxygen-binding prosthetic group of hemoglobin [2,12]. Iron deficiency impairs heme synthesis within erythroid precursor cells, resulting in reduced hemoglobin production and the formation of microcytic hypochromic erythrocytes. Cellular iron homeostasis is tightly regulated by transferrin-mediated transport, ferritin storage, ferroportin export, and hepcidin signaling.

Folate and vitamin B12 are indispensable components of one-carbon metabolism and nucleotide biosynthesis [4,13]. These micronutrients regulate thymidylate and purine synthesis, thereby supporting DNA replication and nuclear maturation in rapidly dividing erythroid progenitor cells. Deficiencies impair cell division and result in ineffective erythropoiesis characterized by megaloblastic changes and macrocytic anemia.

Vitamin B6 is a cofactor of δ-aminolevulinic acid synthase involved in erythropoiesis, which is a rate-limiting enzyme in heme biosynthesis [14]. Deficiency disrupts mitochondrial heme formation and may lead to sideroblastic anemia.

Vitamin A influences erythropoiesis indirectly by modulating iron mobilization from hepatic reserves and regulating erythropoietin expression [15]. Zinc supports cellular proliferation, transcriptional regulation, and erythroid differentiation through zinc-dependent enzymes and transcription factors [16,17]. Copper is equally important for iron transport and utilization, functioning as a cofactor for ceruloplasmin and hephaestin, which facilitate iron oxidation and transferrin loading [18].

Antioxidant micronutrients, such as selenium and vitamin E, safeguard erythrocytes from oxidative damage by regulating glutathione-dependent antioxidant pathways and preventing lipid peroxidation of red cell membranes [19,20]. Deficiencies in these nutrients reduce erythrocyte lifespan and increase susceptibility to hemolysis.

Collectively, these micronutrients function as an integrated metabolic network, and disruption at multiple levels contributes significantly to the development and progression of nutritional anemia. The major micronutrients involved in erythropoiesis, their deficiency-related manifestations, and generally used biomarkers are outlined in Table 1.

**Table 1:** Major micronutrients involved in erythropoiesis and nutritional anemia.

Micronutrient	Key function in erythropoiesis	Key biomarkers	Deficiency manifestations	Reference
Iron (Fe)	Heme synthesis, hemoglobin formation, oxygen transport	↓ serum ferritin; ↓ serum iron; ↑ TIBC; ↓ transferrin saturation	Microcytic hypochromic anemia, fatigue, pallor, pica, koilonychia	[2,3]
Folate (vitamin B9)	One-carbon metabolism, DNA synthesis, erythroid cell division	↓ serum/RBC folate; ↑ homocysteine	Megaloblastic anemia, glossitis, fatigue, elevated homocysteine	[21]
Vitamin B12	DNA synthesis, RBC maturation, myelin maintenance	↓ serum vitamin B12; ↑ methylmalonic acid; ↑ homocysteine	Megaloblastic anemia, pernicious anemia, peripheral neuropathy, cognitive changes, glossitis	[4,22]
Vitamin B6	Cofactor for δ-aminolevulinic acid synthase in heme biosynthesis	↓ plasma pyridoxal-5-phosphate; ↑ erythrocyte protoporphyrin	Sideroblastic anemia, fatigue, irritability, peripheral neuropathy	[23]
Vitamin A	Erythroid differentiation, immune regulation, and iron mobilization from stores	↓ serum retinol; ↓ retinol-binding protein	Anemia, night blindness, impaired immunity, poor growth	[15,24]
Copper (Cu)	Iron transport through ceruloplasmin and hephaestin, iron mobilization, erythropoiesis	↓ serum copper; ↓ ceruloplasmin	Anemia, neutropenia, impaired iron utilization, bone abnormalities, and neurological symptoms	[18,25-27]
Zinc (Zn)	DNA/RNA synthesis, enzyme activity, erythroid cell proliferation, and immune regulation	↓ serum/plasma zinc; reduced alkaline phosphatase activity may support deficiency	Anemia, impaired immunity, growth retardation, poor wound healing, taste changes	[28-30]
Vitamin C	Reduces ferric to ferrous iron, enhances non-heme iron absorption, antioxidant protection	↓ plasma ascorbic acid	Fatigue, poor wound healing, gum bleeding, increased infection risk, impaired iron absorption	[31,32]
Vitamin E	Protects RBC membranes from oxidative damage and lipid peroxidation	↓ serum α-tocopherol; ↑ Markers of lipid peroxidation	Hemolytic anemia, increased RBC fragility, neuromuscular symptoms	[19]
Selenium (Se)	Supports glutathione peroxidase activity and antioxidant defense in erythrocytes	↓ serum/plasma selenium; ↓ glutathione peroxidase activity	Increased oxidative stress, reduced RBC survival, and immune dysfunction	[20,27,33]

Types of nutritional anemia

Nutritional anemia encompasses a heterogeneous group of disorders arising from deficiencies of micronutrients essential for erythropoiesis and red blood cell survival. The hematological presentation varies depending on the specific nutrient deficiency, severity of depletion, and coexistence of multiple deficiencies. The major forms of nutritional anemia can be broadly classified according to red blood cell morphology, underlying nutrient deficiency, and characteristic diagnostic features, as summarized in Table 2.

**Table 2:** Major types of nutritional anemia, RBC morphology, causes, and key features.

Type of anemia	RBC morphology	Key cause	Key features	Reference
Iron deficiency anemia (IDA)	Microcytic, hypochromic RBCs	Iron deficiency due to poor intake, blood loss, malabsorption, or increased demand	Low hemoglobin, low MCV, low serum ferritin, low serum iron, high TIBC, low transferrin saturation	[2,6,34]
Megaloblastic anemia	Macrocytic, oval RBCs; macro-ovalocytes	Folate or vitamin B12 deficiency	High MCV, hypersegmented neutrophils, glossitis; neurological symptoms may occur in vitamin B12 deficiency	[13,35]
Copper deficiency anemia	Variable; may be microcytic, normocytic, or macrocytic	Copper deficiency causes impaired iron transport and utilization	Anemia with neutropenia, low serum copper, low ceruloplasmin, and possible neurological symptoms	[18,25-27,36]
Sideroblastic anemia	Microcytic or dimorphic RBCs; basophilic stippling may be present	Vitamin B6 deficiency, copper deficiency, alcohol, drugs, toxins, or lead toxicity	Ring sideroblasts in bone marrow, impaired heme synthesis, and increased iron accumulation in erythroblasts	[37]
Dimorphic anemia	Mixed microcytic and macrocytic RBC populations	Combined deficiencies, commonly iron with folate or vitamin B12 deficiency	Mixed RBC population, variable MCV, high RDW, features of more than one deficiency	[4,21,22]
Nutritional anemia complicated by inflammation	Usually normocytic, may become microcytic	Chronic inflammation, infection, or inflammatory disease with altered iron metabolism	Normal or high ferritin, low serum iron, low transferrin saturation, increased hepcidin activity	[6,10,38]

Globally, IDA is the most prevalent subtype and is characterized by impaired hemoglobin synthesis resulting from inadequate iron availability. The condition typically presents with microcytic hypochromic erythrocytes, reduced serum ferritin levels, lowered transferrin saturation, and increased total iron-binding capacity [2,12]. Clinically, patients commonly present with fatigue, pallor, exertional dyspnea, pica, and koilonychia.

Folate and vitamin B12 deficiencies result in megaloblastic anemia due to defective DNA synthesis and impaired nuclear maturation of erythroid precursor cells [13]. Peripheral blood findings include macrocytosis, hyper-segmented neutrophils, and anisopoikilocytosis. While both deficiencies produce similar hematological abnormalities, vitamin B12 deficiency is additionally associated with neurological manifestations, including peripheral neuropathy, cognitive dysfunction, and subacute combined degeneration of the spinal cord [22,39].

Vitamin B6 deficiency contributes to sideroblastic anemia through impaired heme biosynthesis and mitochondrial iron accumulation within erythroblasts [37]. Copper deficiency anemia resembles IDA clinically but results primarily from defective iron transport and mobilization.

Deficiencies of vitamin A, zinc, selenium, and vitamin C also contribute to anemia through alterations in iron metabolism, oxidative stress regulation, and erythroid differentiation. In clinical practice, multiple deficiencies frequently coexist, particularly in malnourished populations, resulting in mixed or dimorphic anemia characterized by simultaneous microcytic and macrocytic red blood cell populations.

The coexistence of multiple micronutrient deficiencies complicates both diagnosis and treatment, highlighting the importance of integrated nutritional assessment rather than isolated evaluation of individual nutrients.

Pathophysiology of micronutrient-associated nutritional anemia

**Figure 2:**
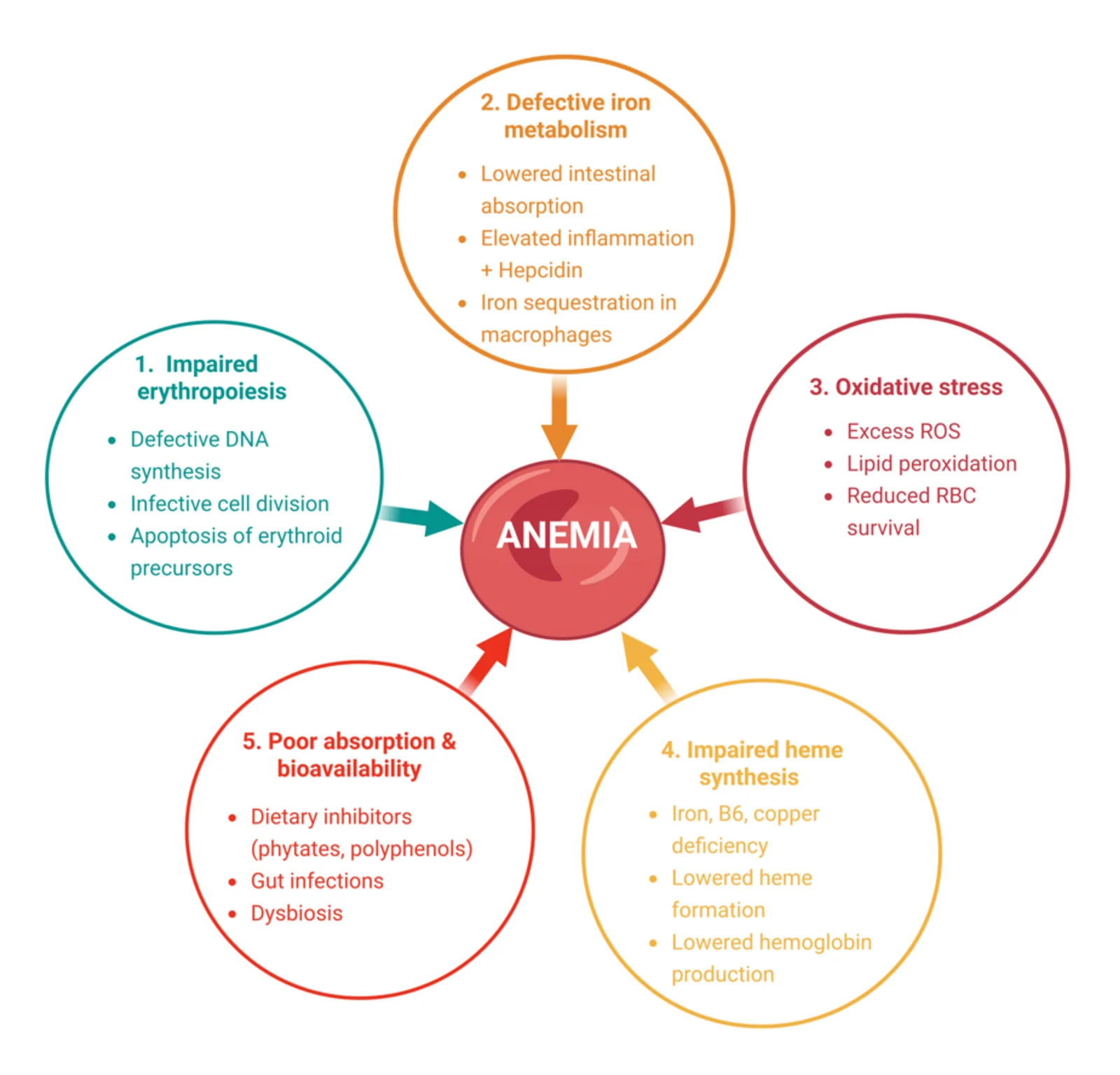
Pathophysiological mechanisms of micronutrient-associated anemia. ROS: reactive oxygen species, RBC: red blood cell. Image Credit: This figure was created using BioRender (BioRender.com, Toronto, ON, Canada).

Figure 2 summarizes the major mechanisms by which micronutrient deficiencies contribute to anemia, including impaired erythropoiesis, defective iron metabolism, impaired heme synthesis, oxidative stress, reduced erythrocyte survival, poor absorption, and inflammation-mediated iron sequestration.

Impaired Erythropoiesis

Erythropoiesis is critically dependent on coordinated micronutrient availability for DNA replication, mitochondrial metabolism, and hemoglobin synthesis. Deficiencies of vitamin B12 and folate disrupt one-carbon metabolism and impair thymidylate synthesis, leading to defective DNA replication and ineffective erythropoiesis [21]. As nuclear maturation becomes delayed relative to cytoplasmic development, erythroid precursor cells enlarge abnormally, producing megaloblastic changes and macrocytic anemia.

Iron deficiency directly limits heme synthesis and hemoglobin formation, thereby impairing erythrocyte maturation and reducing oxygen-carrying capacity. Zinc deficiency further aggravates erythropoietic dysfunction by impairing enzymatic activity, transcriptional regulation, and cellular proliferation within the bone marrow [28].

Recent evidence also implicates mitochondrial dysfunction and oxidative stress in ineffective erythropoiesis. Disruption of mitochondrial iron metabolism affects ATP production, heme biosynthesis, and erythroid cell survival, contributing to anemia progression [40].

Defective Iron Metabolism

Anemia frequently develops not only from absolute iron deficiency but also from dysregulation of systemic iron metabolism. The hepcidin-ferroportin axis stringently regulates Iron homeostasis [41,42]. Inflammatory cytokines, particularly interleukin-6, stimulate hepatic hepcidin synthesis, which inhibits ferroportin-mediated iron export from enterocytes and macrophages [28,40,43,44]. Consequently, iron becomes sequestered within storage tissues, producing functional iron deficiency despite adequate iron reserves.

Vitamin A deficiency further impairs iron mobilization from hepatic stores, while copper deficiency disrupts ceruloplasmin-dependent oxidation of ferrous iron required for transferrin binding and systemic transport [15,25,26]. Excessive zinc intake may indirectly impair iron metabolism by reducing copper absorption [28,29].

These findings suggest that many cases of nutritional anemia involve impaired iron use and transport, not only reduced iron intake or depleted stores.

Oxidative Stress and Red Blood Cell Survival

Red blood cells are continuously exposed to oxygen and iron-mediated reactive oxygen species (ROS), rendering them susceptible to oxidative damage. Antioxidant micronutrients, such as selenium and vitamin E, protect erythrocytes by regulating glutathione-dependent antioxidant pathways and preventing lipid peroxidation [20,33].

Deficiency of antioxidant micronutrients increases membrane instability, hemoglobin oxidation, and erythrocyte fragility, ultimately shortening red blood cell lifespan. Oxidative stress is further amplified during chronic inflammation, infection, and combined micronutrient deficiencies.

Gut Absorption and Micronutrient Transport

Micronutrient bioavailability depends heavily on intestinal absorption and transporter-mediated cellular uptake. Iron absorption involves divalent metal transporter-1 (DMT1), ferroportin, and hephaestin, whereas zinc transport is mediated by ZIP and ZnT transporter families [30].

Malabsorption disorders, such as inflammatory bowel disease, celiac disease, and post-bariatric surgery syndromes, significantly impair micronutrient uptake [43]. Upcoming studies also showcase the role of the gut microbiome in regulating micronutrient metabolism, inflammation, and iron bioavailability.

Collectively, these mechanisms show that nutritional anemia is shaped by nutrient intake, intestinal absorption, inflammation, micronutrient transport, and red blood cell survival.

Micronutrient interactions

**Figure 3:**
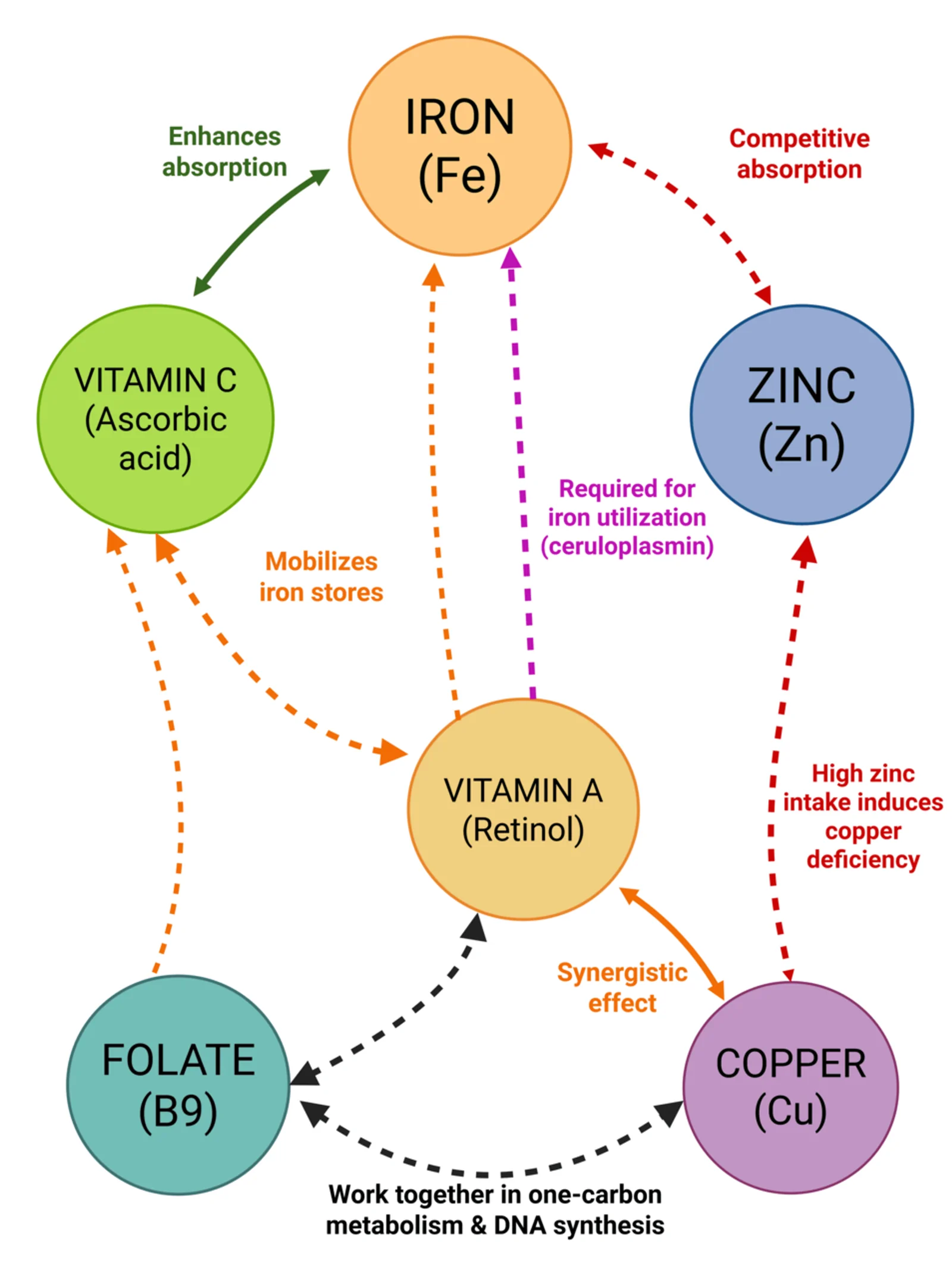
Interactions among micronutrients involved in nutritional anemia. This figure represents key micronutrient interactions relevant to anemia, including the enhancing effect of vitamin C on iron absorption, the role of vitamin A in iron mobilization, competitive interactions between iron and zinc, zinc-induced copper deficiency, and the coordinated role of folate and copper in erythropoiesis-related pathways. Image Credit: This figure was created using BioRender (BioRender.com, Toronto, ON, Canada).

Micronutrients involved in erythropoiesis and iron metabolism interact extensively at the levels of absorption, transport, storage, and cellular utilization. These interactions may be synergistic or antagonistic and significantly influence anemia pathogenesis and therapeutic response (Figure 3).

Iron and zinc share common intestinal transport pathways, particularly divalent metal transporter-1, leading to competitive inhibition when consumed in disproportionate quantities [28,29]. High-dose iron supplementation may reduce zinc absorption, whereas excessive zinc intake may impair iron uptake.

Zinc-induced copper deficiency represents another clinically significant interaction [25,26]. Elevated zinc levels stimulate intestinal metallothionein synthesis, which selectively binds copper and lowers systemic absorption. Copper deficiency subsequently impairs ceruloplasmin activity and iron mobilization, resulting in anemia resembling iron deficiency [23].

Conversely, several micronutrients exhibit synergistic relationships. Vitamin C enhances non-heme iron absorption by reducing ferric (Fe^+3^) iron to the more bioavailable ferrous (Fe^+2^) form and negating the negative effects of phytates and polyphenols [31,32]. Vitamin A improves iron mobilization from storage tissues, while zinc facilitates vitamin A transport through retinol-binding protein synthesis.

These interactions also show why supplementation should be planned carefully. Although micronutrient replacement is essential in deficiency states, excessive or poorly balanced supplementation may hinder the absorption or utilization of other nutrients. High zinc intake, for instance, may contribute to copper deficiency, while large doses of iron may affect zinc absorption in some settings. Similarly, providing folate without identifying vitamin B12 deficiency may improve blood parameters while allowing neurological complications to progress. For this reason, nutrient interactions should be considered during both clinical treatment and public health supplementation programs [25,26,28,29]. Important synergistic, antagonistic, and inhibitory interactions among micronutrients and dietary factors relevant to nutritional anemia are outlined in Table 3.

**Table 3:** Major micronutrient interactions influencing nutritional anemia.

Micronutrient interaction	Type of interaction	Mechanism	Clinical implication	References
Vitamin C + iron	Synergistic	Vitamin C reduces ferric iron to ferrous iron and improves non-heme iron absorption	Improves response to iron therapy, especially in plant-based diets	[31,32]
Vitamin A + iron	Synergistic	Vitamin A supports iron mobilization from hepatic stores and may improve erythropoiesis	Vitamin A deficiency may worsen anemia despite adequate iron intake	[15,24]
Iron + zinc	Competitive	Iron and zinc may compete for intestinal absorption pathways, especially at high supplemental doses	Excessive iron supplementation may reduce zinc absorption	[29,30]
Zinc + copper	Antagonistic	High zinc intake increases intestinal metallothionein, which binds copper and reduces copper absorption	Can cause copper deficiency anemia, neutropenia, and impaired iron utilization	[26,27]
Copper + iron	Synergistic	Copper-dependent ceruloplasmin and hephaestin help oxidize iron for transferrin binding and transport	Copper deficiency may mimic iron deficiency anemia	[26,36]
Folate + vitamin B12	Synergistic	Both are required for one-carbon metabolism and DNA synthesis in erythroid precursors	Combined deficiency causes megaloblastic anemia; folate may mask B12-related neurological symptoms	[21]
Vitamin E + selenium	Synergistic	Both support antioxidant defense and protect RBC membranes from oxidative damage	Deficiency may increase RBC fragility and reduce erythrocyte survival	[19,33]
Phytates + iron/zinc	Inhibitory dietary interaction	Phytates bind minerals in the gut and reduce absorption	High-phytate diets may worsen iron and zinc deficiency	[45]
Polyphenols/tannins + iron	Inhibitory dietary interaction	Tea, coffee, and some plant polyphenols form insoluble complexes with non-heme iron	Reduces iron absorption when consumed with meals	[46]
Calcium + iron	Competitive/inhibitory	High calcium intake may reduce iron absorption when taken together	Iron and calcium supplements may need separate timing	[47]

These interactions highlight the limitations of isolated supplementation strategies and support the growing need for integrated multi-micronutrient therapeutic approaches in nutritional anemia.

Dietary and environmental factors

Dietary composition strongly influences micronutrient bioavailability and plays a central role in the development of nutritional anemia (Table 4). In many low- and middle-income countries, diets are dominated by inexpensive staple cereals or tubers and contain relatively small quantities of fruits, vegetables, pulses, and animal-source foods. Although staple-based diets may provide adequate energy, they can have low micronutrient density and limited dietary diversity. This dietary pattern increases the likelihood of inadequate intake of iron, folate, vitamin B12, and vitamin A, particularly during periods of increased physiological demand such as childhood, adolescence, menstruation, pregnancy, and lactation [48-50].

**Table 4:** Dietary factors that influence iron absorption.

Section	Item/factor	Details/examples	Mechanism/effect
Enhancers of iron absorption	Citrus fruits	Lemon, orange and other vitamin C-rich fruits	Reduces Fe³⁺ to Fe²⁺, forms soluble complexes, and enhances iron absorption
	Meat, fish, poultry	Animal-source foods	Improves iron absorption
	Fermented foods	Fermented food products	Enhances iron bioavailability
	Sprouting and soaking	Sprouted or soaked grains/legumes	Helps improve mineral availability
	Cast iron cookware	Cooking in iron utensils	May increase dietary iron content
Inhibitors of iron absorption	Phytates	Whole grains, legumes	Binds iron and forms insoluble complexes, decreasing absorption
	Polyphenols	Tea, coffee	Reduces iron absorption
	Excess calcium	High calcium intake/foods	May inhibit iron absorption
	Tannins	Tea and tannin-containing foods/drinks	Decreases iron absorption
	Processed foods	Processed and fast foods	May negatively affect micronutrient status

Plant-based diets, although nutritionally valuable, predominantly contain non-heme iron, which has relatively low bioavailability in comparison to heme iron derived from animal-based diets. Furthermore, cereals and legumes contain phytates that chelate iron and zinc, reducing their intestinal absorption [45].

Polyphenols and tannins from coffee, tea, and certain plant foods similarly inhibit iron absorption by forming insoluble complexes within the gastrointestinal tract [46]. Traditional food-processing practices like soaking, germination, and fermentation can reduce anti-nutritional factors and improve mineral bioavailability.

Protein-energy malnutrition further contributes to anemia by impairing the synthesis of transport proteins, including transferrin and retinol-binding protein [51]. Socioeconomic factors, such as food insecurity, poor sanitation, limited dietary diversity, and inadequate maternal nutrition, significantly increase susceptibility to micronutrient deficiencies.

Infectious diseases and chronic inflammation also play major roles in anemia pathogenesis [34,52]. Helminthic infestations cause chronic blood loss, while malaria promotes erythrocyte destruction and suppresses erythropoiesis [53]. Chronic inflammatory states induce hepcidin-mediated iron sequestration, reducing iron availability despite adequate stores.

Recent studies additionally suggest that climate-related agricultural changes and soil micronutrient depletion may contribute to declining nutritional quality of staple foods, thereby influencing anemia prevalence at the population level [54].

Clinical implications

Micronutrient-associated anemia has significant systemic consequences extending beyond impaired oxygen transport. Chronic anemia adversely affects physical performance, cognitive function, immune competence, pregnancy outcomes, and quality of life.

Mixed micronutrient deficiencies frequently produce atypical hematological presentations that complicate diagnosis. For example, coexisting iron deficiency may mask macrocytosis in vitamin B12 deficiency, resulting in apparently normocytic anemia despite severe underlying pathology [13,35].

Pregnant women are particularly vulnerable due to increased demands for iron, folate, and vitamin B12 during fetal growth and placental development. Maternal anemia is known to be associated with premature birth and lower birth weight, and affects neural development as well as cognitive functions [55].

Children with micronutrient deficiencies may exhibit impaired cognitive development, reduced academic performance, and compromised immune function [56]. Elderly individuals are similarly vulnerable due to poor dietary intake, chronic disease, and impaired gastrointestinal absorption [57].

Subclinical deficiencies represent an additional clinical challenge. Individuals may maintain normal hemoglobin concentrations despite depleted micronutrient stores, thereby remaining undiagnosed until physiological stress or disease progression precipitates overt anemia.

These observations emphasize the need for early detection and comprehensive micronutrient assessment in vulnerable populations.

Diagnostic challenges

Diagnosis of nutritional anemia remains formidable due to clinical indications that are often nonspecific, and multiple micronutrient deficiencies commonly coexist. Symptoms such as fatigue, weakness, pallor, and reduced exercise tolerance occur across various anemia subtypes and lack diagnostic specificity.

Conventional laboratory evaluation typically relies on hemoglobin concentration, mean corpuscular volume, serum ferritin, transferrin saturation, and vitamin B12 levels. However, these biomarkers are influenced by inflammation, infection, liver disease, and metabolic stress. Ferritin, for instance, functions as an acute-phase reactant and may appear elevated despite underlying iron deficiency [42,45].

Mixed deficiency states further complicate interpretation. Concurrent iron deficiency may normalize mean corpuscular volume in vitamin B12 deficiency, thereby masking macrocytic changes. Similarly, inflammatory conditions alter iron trafficking and obscure the assessment of true iron status.

Recent advances in diagnostics include soluble transferrin receptor assays, reticulocyte hemoglobin content, hepcidin measurements, methylmalonic acid assessment, and homocysteine profiling. Emerging omics-based approaches, including proteomics and metabolomics, may further improve the detection of early and subclinical micronutrient deficiencies [20,58].

In clinical practice, anemia-related biomarkers should be interpreted in relation to inflammatory status and overall nutritional condition. Ferritin is useful for assessing iron stores, but it may be falsely elevated during infection, inflammation, liver disease, or chronic illness. Measurement of inflammatory indicators, such as C-reactive protein, may assist in separating absolute iron deficiency from inflammation-associated functional iron insufficiency. Reticulocyte hemoglobin content and soluble transferrin receptor can provide additional information on iron availability for erythropoiesis, while methylmalonic acid and homocysteine may help differentiate vitamin B12 deficiency from folate deficiency. Therefore, a combined diagnostic approach is more reliable than relying solely on hemoglobin concentration, mean corpuscular volume, or ferritin [20,42,46,59].

**Figure 4:**
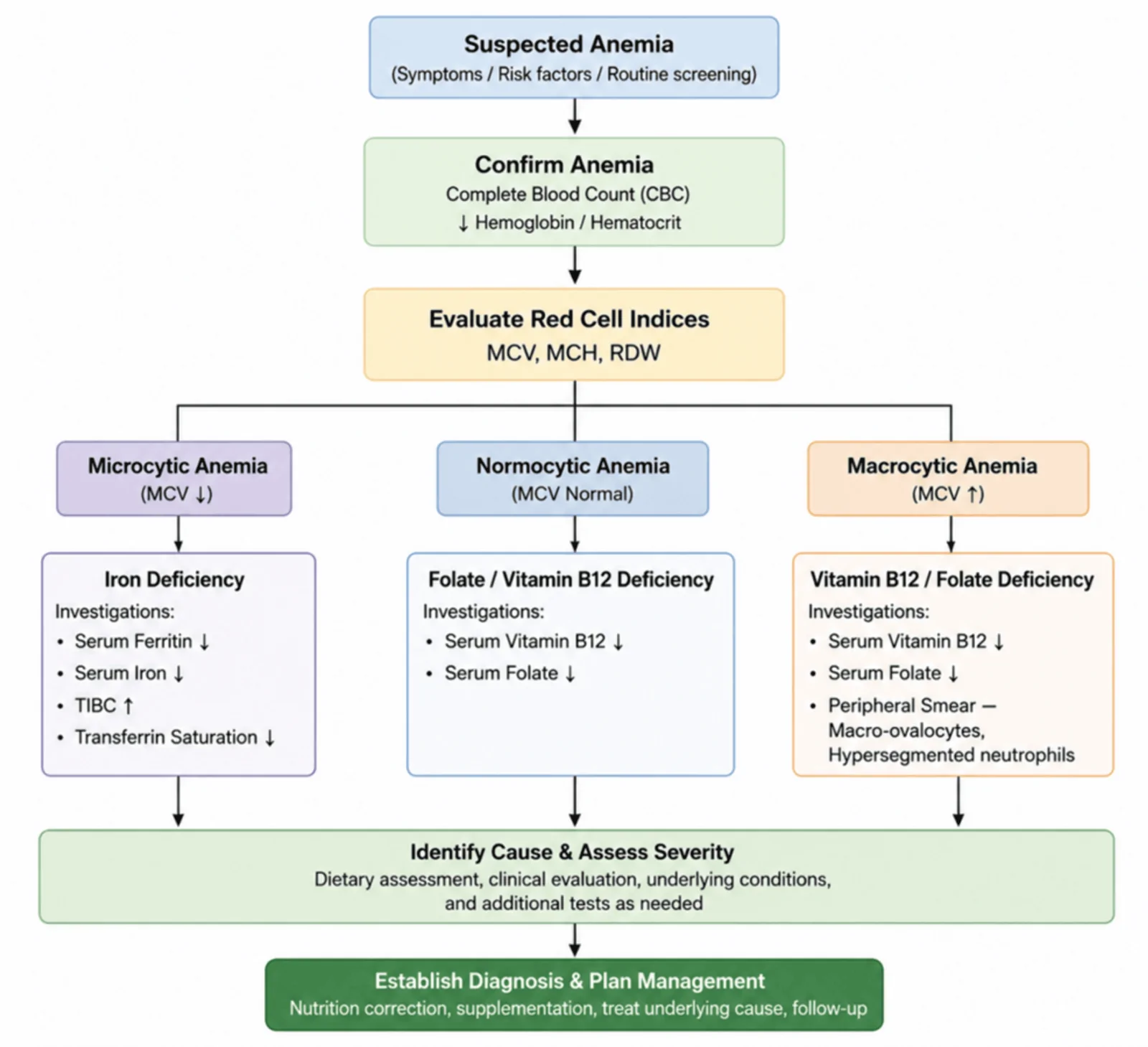
Diagnostic approach to nutritional anemia. This flowchart outlines a stepwise diagnostic approach for nutritional anemia, beginning with clinical assessment and complete blood count, followed by evaluation of red blood cell indices, classification into microcytic, normocytic, or macrocytic anemia, micronutrient testing, and integrated interpretation for diagnosis and management. MCV: mean corpuscular volume, MCH: mean corpuscular hemoglobin, RDW: red cell distribution width, TIBC: total iron-binding capacity. Image Credit: This figure was created using BioRender (BioRender.com, Toronto, ON, Canada).

Integrated multi-marker diagnostic strategies are increasingly recognized as essential for accurate characterization of nutritional anemia and individualized therapeutic planning, as depicted in Figure 4.

Management strategies

Traditional management of nutritional anemia has focused primarily on iron supplementation. However, cumulative evidence indicates that isolated iron supplementation is frequently insufficient because multiple micronutrient deficiencies commonly coexist.

Multi-micronutrient supplementation strategies incorporating iron, folate, zinc, vitamin B12, and A have demonstrated superior improvements in hemoglobin concentration, erythropoietic recovery, and overall nutritional status compared with iron monotherapy [60,61].

However, the response to multi-micronutrient supplementation may vary across individuals and populations. Factors such as baseline nutritional status, inflammation, infection burden, dietary pattern, pregnancy status, gastrointestinal absorption, and adherence to therapy can strongly influence treatment outcomes. In addition, supplementation without proper assessment may sometimes create nutrient imbalances. For example, excessive zinc intake can reduce copper absorption, while iron supplementation may be less effective when inflammation increases hepcidin activity and restricts iron availability. Therefore, whenever possible, supplementation should be guided by clinical evaluation, dietary history, and relevant biochemical markers instead of relying only on a uniform treatment approach [25,26,41,42,60,61].

Dietary diversification remains a cornerstone of anemia prevention and management. Increased consumption of foods rich in micronutrients, including green leafy vegetables, legumes, fruits, dairy products, and animal-derived proteins, can significantly improve nutritional status. Food-processing techniques, such as fermentation and germination, may increase mineral bioavailability by decreasing phytate content.

Biofortification of staple crops with provitamin A, zinc, and iron represents a sustainable population-level strategy for defending against micronutrient deficiencies [62]. At the clinical level, management should additionally address underlying conditions, including chronic inflammation, infections, gastrointestinal disease, and malabsorption syndromes.

Personalized nutrition approaches based on biomarker-guided supplementation and individual nutritional profiling are emerging as promising strategies for enhancing therapeutic efficacy and minimizing adverse interactions among micronutrients. 

**Figure 5:**
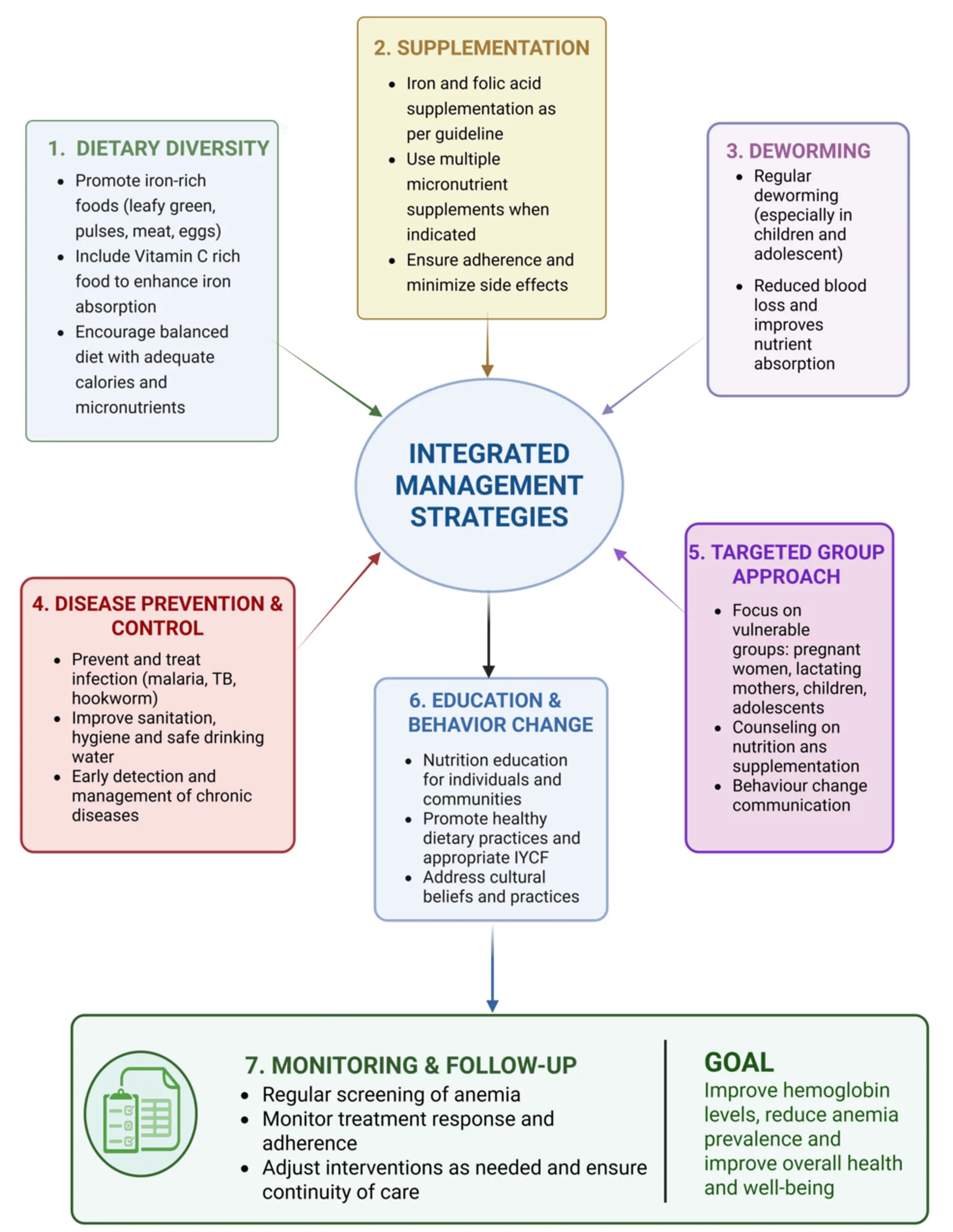
Integrated management strategies for nutritional anemia. This figure presents a comprehensive management framework for nutritional anemia, integrating dietary diversification, micronutrient supplementation, deworming, infection control, targeted interventions for vulnerable populations, education, behavior change, and monitoring of treatment response. Image Credit: This figure was created using BioRender (BioRender.com, Toronto, ON, Canada).

A comprehensive management framework for nutritional anemia is depicted in Figure 5.

Public health perspective

Nutritional anemia remains a major global public health concern despite decades of supplementation and fortification programs. Most current public health interventions continue to focus predominantly on iron deficiency, often overlooking the contribution of multiple interacting micronutrient deficiencies.

Large-scale programs, including iron-folic acid (IFA) supplementation, diversification of diet, and food fortification, have shown measurable benefits; however, implementation challenges, such as poor compliance, inadequate healthcare access, gastrointestinal side effects, and limited dietary awareness, continue to reduce program effectiveness.

In India, national initiatives, including the National Iron Plus Initiative (NIPI) and Anemia Mukt Bharat (AMB), have improved awareness and screening coverage, yet anemia prevalence remains high, particularly in women and children [63-66]. Although these interventions have improved access to preventive measures, their impact is often limited by poor adherence due to gastrointestinal side effects, irregular supplement supply, inadequate counselling, and low compliance among adolescents and pregnant women [66,67]. Furthermore, a considerable proportion of anemia in India is attributable not only to iron deficiency but also to deficiencies of vitamin B12, folate, and other micronutrients, alongside recurrent infections, parasitic infestations, hemoglobinopathies, chronic inflammation, and poor maternal nutrition, which are not adequately addressed through iron supplementation alone [3,9,68]. The effectiveness of food fortification is further constrained by India's diverse dietary practices, as many rural and tribal communities depend on locally milled cereals and traditional diets that often fall outside centralized fortification systems. In addition, cereal-based diets with low dietary diversity, high phytate content, poor sanitation, and socioeconomic inequalities continue to reduce micronutrient bioavailability and perpetuate anemia [3,64]. In some communities, limited intake of animal-source foods may also contribute to low bioavailability of iron, vitamin B12, and other micronutrients. Among adolescent girls and women of reproductive age, menstrual blood loss, pregnancy, repeated childbirth, and inadequate dietary intake further increase the risk of anemia. Parasitic infections, poor sanitation, food insecurity, and limited access to early diagnosis may add to the burden, particularly in vulnerable populations [63-65].

Therefore, anemia control strategies in India need to extend beyond IFA supplementation alone. A more comprehensive approach should include dietary diversification, nutrition education, deworming, infection control, improved sanitation, food fortification, and screening for vitamin B12, folate, and other micronutrient deficiencies where clinically indicated. Also, a region-specific, integrated approach is required. Such an approach may improve the effectiveness of existing public health programs and help address mixed-deficiency anemia more efficiently.

Future public health interventions should incorporate nutrition education, infection control, agricultural sustainability, food security initiatives, and maternal-child healthcare services. Multi-sectoral collaboration involving healthcare, agriculture, education, and policy systems is essential for sustainable reduction of nutritional anemia burden.

Emerging research areas

Recent advances in molecular nutrition and systems biology have significantly expanded the understanding of nutritional anemia [69,70]. Potential data indicate that the gut microbiome has a key role in regulating micronutrient absorption, systemic inflammation, and iron homeostasis.

Nutrigenomics and epigenetic research have further demonstrated that genetic variability influences individual responses to micronutrient intake, absorption, and supplementation. Variations in genes regulating iron transport, hepcidin expression, and folate metabolism may contribute to inter-individual differences in anemia susceptibility and treatment response.

Recent advances in omics technologies have opened new avenues for understanding the complex molecular basis of micronutrient-deficiency anemia. Integrated approaches such as genomics, transcriptomics, proteomics, and metabolomics enable the comprehensive identification of genes, proteins, and metabolites associated with micronutrient status, erythropoiesis, and iron homeostasis. These technologies have the potential to identify novel biomarkers for the early detection of micronutrient deficiencies, distinguish between different types of anemia, and facilitate personalized nutritional interventions. As multi-omics data become increasingly integrated with clinical and nutritional information, they are expected to improve diagnostic precision and provide deeper insights into the molecular mechanisms underlying anemia, paving the way for more targeted and effective prevention and treatment strategies [71,72].

Artificial intelligence and machine learning may also help analyze large biomarker datasets and identify individuals at higher risk of micronutrient deficiencies.

Additionally, increasing attention is being directed toward the relationship between micronutrient imbalance, oxidative stress, inflammation, and cancer-associated anemia, representing an important area for future translational research [24].

Future directions

**Figure 6:**
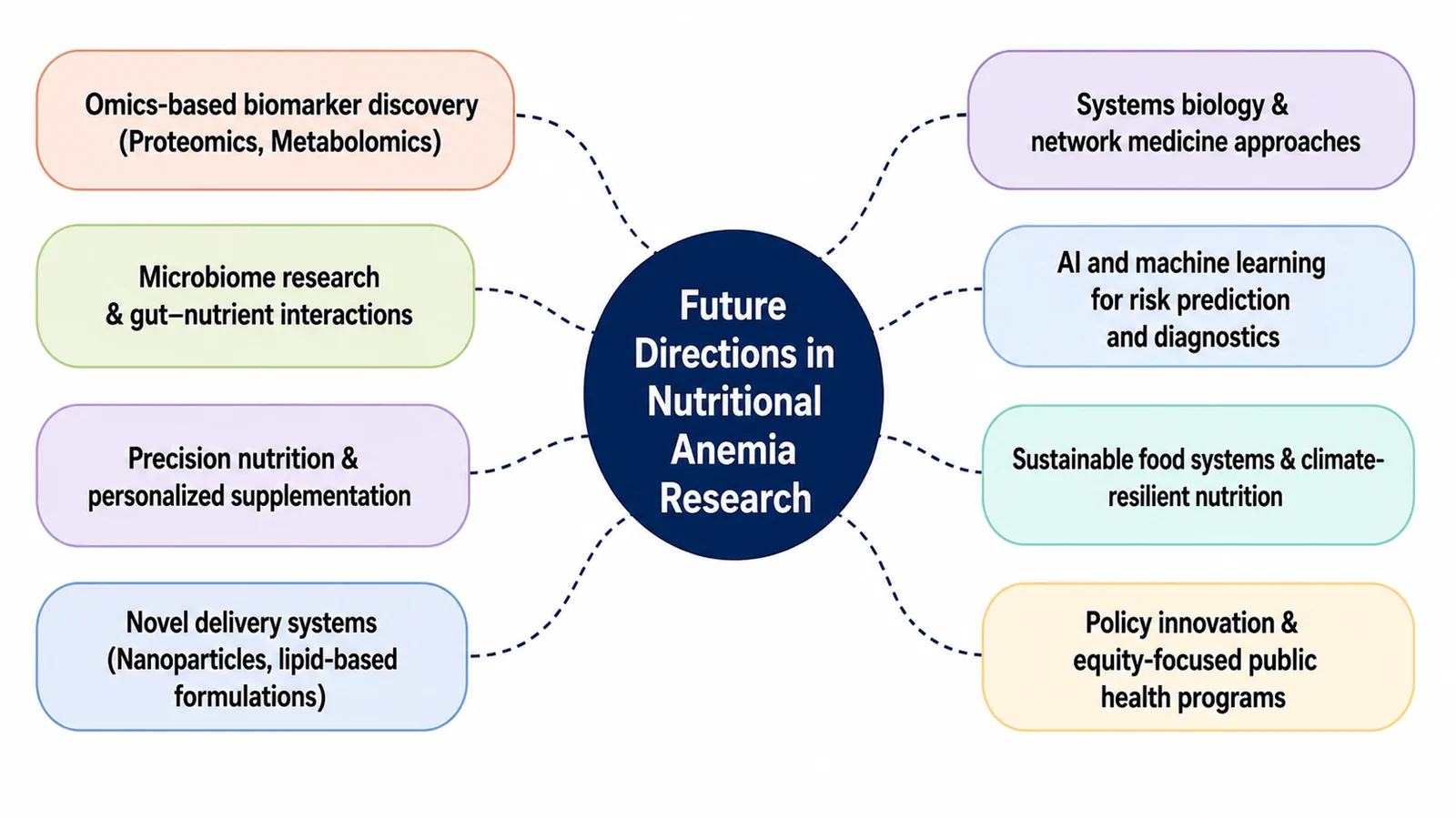
Emerging research areas and future directions in nutritional anemia. This figure highlights future research priorities in nutritional anemia, including omics-based biomarker discovery, microbiome research, precision nutrition, novel delivery systems, systems biology, artificial intelligence-based risk prediction, sustainable food systems, and equity-focused public health programs. Image Credit: This figure was created using BioRender (BioRender.com, Toronto, ON, Canada).

Future strategies for managing nutritional anemia should transition from isolated nutrient replacement toward integrated systems-based nutritional medicine (Figure 6). Development of multi-micronutrient diagnostic platforms capable of detecting mixed and subclinical deficiencies will be essential for improving early diagnosis.

Personalized nutrition approaches integrating genomic, metabolomic, and proteomic profiling may facilitate individualized supplementation strategies tailored to specific metabolic and inflammatory states. Advances in artificial intelligence and machine learning may further support predictive diagnostics and personalized therapeutic planning.

Greater emphasis should also be placed on microbiome-targeted interventions, biofortified crops, sustainable food systems, and climate-resilient nutritional policies.

Importantly, future research should investigate the interplay between micronutrient deficiencies, oxidative stress, chronic inflammation, and cancer biology, as these interconnected mechanisms may contribute significantly to disease progression and treatment outcomes.

Limitations of the review

This review has certain limitations. As a narrative review, it does not include formal meta-analysis or quantitative grading of evidence. The available literature on some micronutrients, particularly selenium, vitamin E, zinc, and copper, remains variable across different populations and clinical settings. In addition, many studies focus on single-nutrient deficiencies, whereas nutritional anemia in real-world settings often involves overlapping deficiencies, inflammation, infection, poor dietary diversity, and impaired absorption.

Another limitation is that the diagnostic and therapeutic relevance of several emerging biomarkers and omics-based approaches is still evolving. Although these tools may improve early detection and individualized management in the future, their routine use remains limited in many clinical and public health settings. Further population-based studies, longitudinal research, and intervention trials are required to clarify micronutrient interactions and develop practical strategies for managing mixed-deficiency anemia.

## Conclusions

Nutritional anemia is not only an outcome of iron deficiency but also reflects the combined influence of multiple micronutrient deficiencies, inflammation, oxidative stress, impaired absorption, and altered iron utilization. Micronutrients such as vitamin B12, B9 (folate), B6, A, C, E, copper, zinc, and selenium contribute to red blood cell production, heme synthesis, iron transport, antioxidant defense, and erythrocyte survival. When these deficiencies occur together, they can produce mixed hematological features and reduce the effectiveness of conventional iron-centered treatment.

A broader diagnostic and therapeutic approach is therefore needed. Assessment of nutritional anemia should consider dietary history, inflammatory status, red blood cell indices, iron markers, and relevant micronutrient biomarkers. Management should combine dietary diversification, targeted supplementation, treatment of infections and inflammation, food fortification, public health education, and monitoring of treatment response. Future strategies should move toward integrated and individualized anemia care. Advances in biomarker discovery, omics technologies, microbiome research, and precision nutrition may help identify mixed deficiencies earlier and guide more effective interventions. Distinguishing nutritional anemia as a multi-micronutrient disorder may improve clinical care and support stronger public health strategies, particularly for vulnerable groups.
